# A Novel De Novo Mutation of the DHX30 Gene in a Patient With Neurodevelopmental Disorder, Severe Motor Impairment, and Absent Language (NEDMIAL)

**DOI:** 10.7759/cureus.33682

**Published:** 2023-01-12

**Authors:** Mohammad M Alomaim, Aziza M Mushiba

**Affiliations:** 1 College of Medicine, King Saud Bin Abdulaziz University for Health Sciences, Riyadh, SAU; 2 General Pediatrics, King Fahad Medical City, Riyadh, SAU

**Keywords:** pro796leu, nedmial, atp-dependent rna helicase, whole exome sequence, dhx30

## Abstract

Introduction: DExH-Box Helicase 30 (DHX30) is a gene that codes for proteins. It belongs to the class of RNA secondary structure unwinding helicases known as DExH-boxes. There have been numerous reports of pathogenic DHX30 variants. Most mutations, but not all, result in severe phenotypic abnormalities. The most common symptoms are severe motor developmental delay, intellectual disability, sleep disturbances, autism spectrum disorder, seizures, and gait abnormalities.

Objective: The objectives of reporting this case are: To report a novel mutation giving rise to NEDMIAL and to update the literature regarding the manifestation of the case of a rare condition (NEDMIAL).

Case presentation: We report the case of a 12-year-old female who presented with similar complaints of severe motor impairment, seizures, intellectual disability, and absent language and was later diagnosed on Next-Generation Sequencing (NGS) with an autosomal dominant neurodevelopmental disorder (NEDMIAL).

Conclusion: We report a case of neurodevelopmental disorder with severe motor impairment and absent language (NEDMIAL) with a De novo novel DHX30 mutation (p.Pro796Leu) detected by whole exome sequence. We suggest upgrading the variant classification of DHX30:p.Pro796Leu to likely pathogenic, according to the evidence found in our patient. To the best of our knowledge, this is the first reported case of this mutation and disorder in the Middle East.

## Introduction

The gene DHX30 codes for proteins. It is a member of the group of helicases called DExH-boxes that unwind RNA secondary structures [1]. The DHX30 gene is also found to regulate mitochondrial ribosome assembly and various stages of the RNA life cycle [2,3]. Numerous pathogenic DHX30 variants have been reported [1,4]. Mutations that cause loss-of-function mutations in the DHX30 gene result in a milder phenotype, while mutations in helical core motifs (HCM) in the DHX30 gene induce a severe phenotype characterized by intellectual disability, seizures, gait problems, speech impairment, and developmental delay [5]. We present a case, which to the best of our knowledge, is the first reported case of NEDMIAL in the Middle East and the first case with p.(Pro796Leu) mutation in the DHX30 gene.

## Case presentation

**Figure 1:**
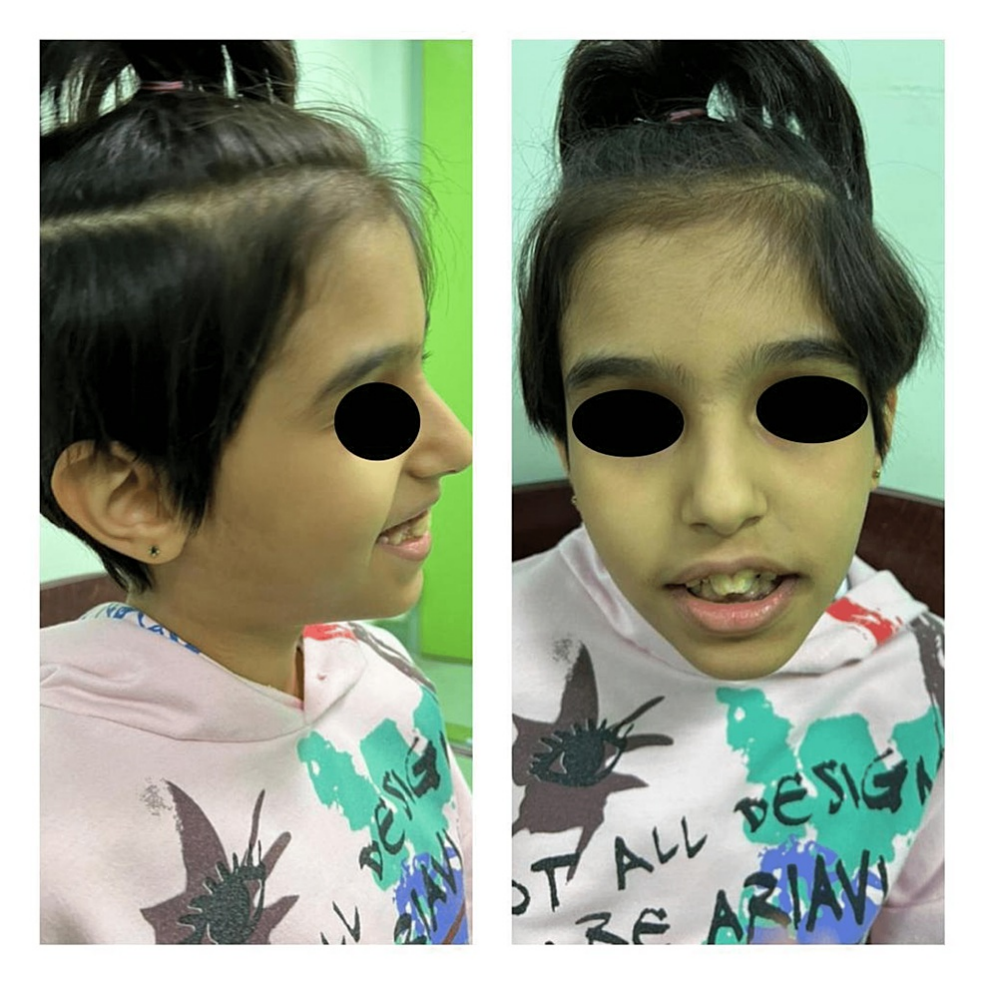
Frontal and lateral view of the face showing the following facial dysmorphism: Synophrys, full lips, high-arched palate, everted lower lip, hairy forehead, overlapping teeth, and prominent central incisors.

A 12-year-old female presented with complaints of seizure disorder, nystagmus, severe receptive and expressive language delays, severe intellectual disability, and stereotypic behavior. There was also a presentation of anemia, although it was not diagnosed later on in the examination and lab workup. The patient had settled dysmorphic features (a low-set ear and a small jaw), synophrys, microcephaly, and a high-arched palate (Figure 1). 

**Figure 2:**
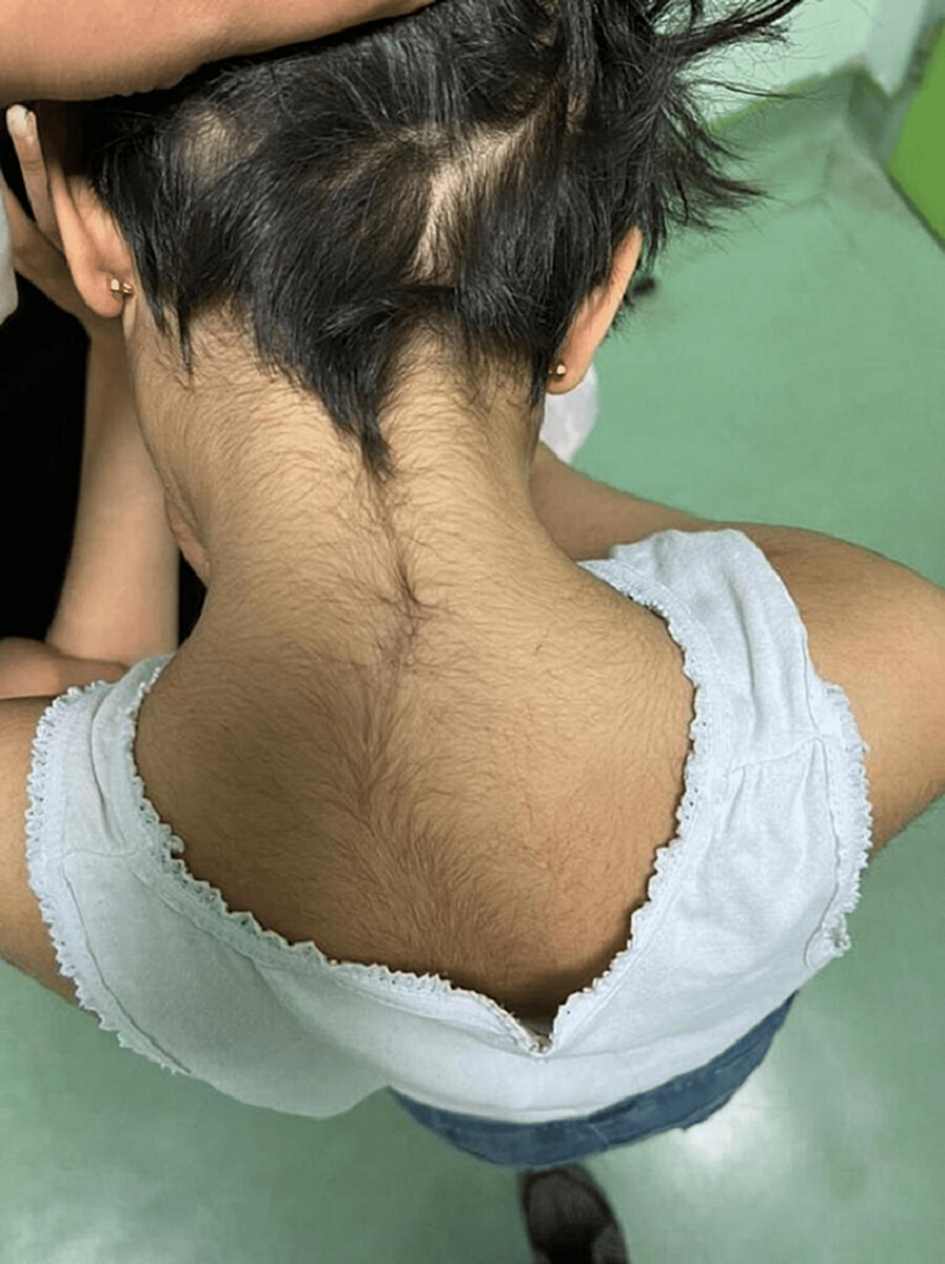
Back of the neck shows a low hairline and hairy back of the neck (hirsutism).

**Figure 3:**
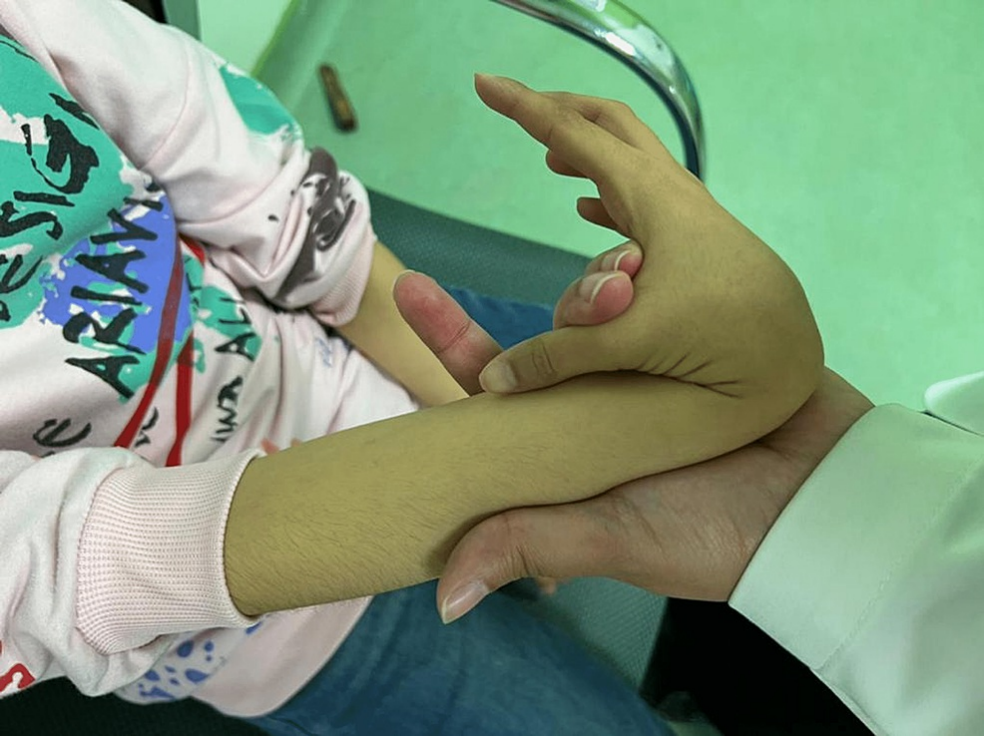
Joints of the hand showing hyperlaxity.

The patient also had hirsutism on the back with a low hairline (Figure 2), skin laxity (Figure 3), hypotonia, and lower limb eczema. 

**Figure 4:**
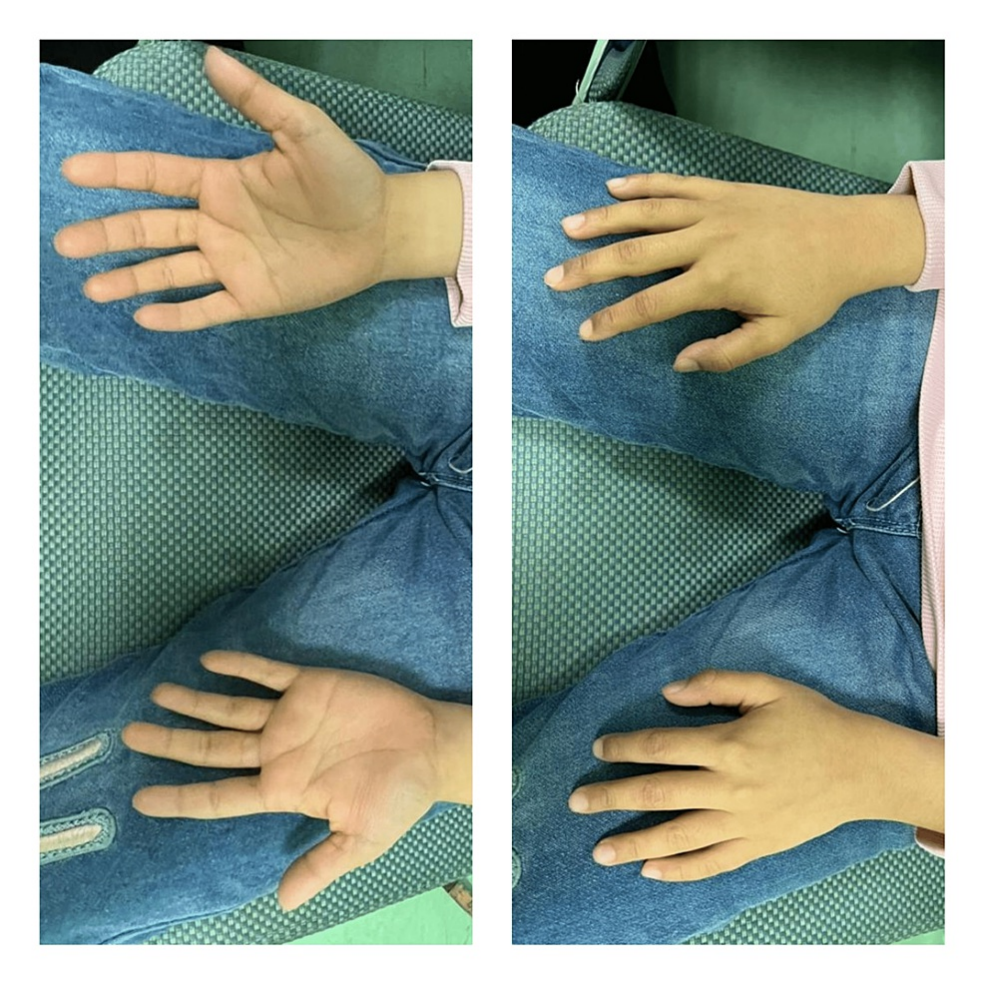
Palmar and dorsal views of the hand showing 5th finger clinodactyly.

The patient was full-term and delivered by cesarean section. She had hypoxic-ischemic encephalopathy at birth and was thus admitted to the Neonatal Intensive Care Unit for a week. Due to fetal distress and oligohydramnios, she had a low birth weight of 1.5 kilograms and 5^th^ finger clinodactyly (Figure 4).

She was also cyanosed at the time of birth. Additionally, she was diagnosed with pulmonary stenosis, for which the patient underwent surgery at the age of five months. The parents also mentioned chest tube insertion. The patient had also had nystagmus since birth. The patient developed seizures at the age of six years. She was started on Levitericitam, but her mother stopped its administration after only a month. The patient also had a history of frequent blood transfusions (about six times until the time of her presentation in our hospital). Each blood transfusion was associated with a spike in fever (transfusion pyrexia). The patient was vaccinated up to age. The patient was the first child of a consanguinity marriage (1st-degree cousins). She had three younger, healthy siblings. Further investigation found that no one else in the family had a neurological or genetic issue.

The patient had frequent mixed seizures (focal and generalized); her most recent seizure occurred a day before her presentation in our hospital. The parents denied any history of neonatal death, abortion, genetic or metabolic diseases, hematological diseases, autism, intellectual disability, or a similar condition in the family. Her weight was in the third percentile, her height was two big squares below the third percentile, and her head circumference was also in the third percentile. On physical examination, three well-circumscribed hyperpigmented lesions on the abdomen and left thigh were discovered. However, there were no hypopigmented skin lesions. The patient did not make any eye contact and was not interactive. She didn't respond to her name. She had a lot of lateral gazes, and another noticeable feature was her finger mannerism and flapping hand movements. The patient had unusual facial grimacing. Furthermore, she had a high tolerance for pain and showed extreme distress when exposed to sounds. Excessive mouthing was another noticeable feature on examination. She had a high-risk M-CHAT score of 16.

The patient had delayed development of motor milestones. She was able to sit without support at the age of one year. She walked alone at the age of two years. At the time of the presentation, she can run and go upstairs and downstairs with rails, but she cannot jump, ride a bike, or walk backward, implying severely delayed fine motor activity. Only the palmar grasp reflex was present (no pincer grasp reflex). Her parents revealed that she couldn't draw or copy crude lines, vertical lines, circles, or triangles and that she could neither feed herself nor dress or undress. She was still in diapers and totally dependent on her mother for every single daily routine activity. The parents also revealed that she said her first word (MAMA) when she was eight years old and hasn't spoken another word or sentence since.

Her expressive language, adaptive skills, and receptive language were severely delayed. Only sometimes would she display repetitive vocalization. According to the medical team's plan, the patient was sent to audiology for a hearing evaluation to rule out any hearing problems that could have caused speech abnormalities. Upon inquiry, it was found that there was no family history of hearing loss. The otoscopic examination was unremarkable for both the left and right ears. Tympanometry (226 Hz) revealed normal middle ear pressure, static compliance, and ear canal volume on both sides. Ipsilateral middle ear muscle reflexes (500 Hz, 1000 Hz, and 2000 Hz) were present at expected sensation levels bilaterally. Distortion-product otoacoustic emissions (2000-8000 Hz) were present and robust on both sides, which was suggestive of normal outer hair cell function. Thus, the results indicated normal hearing bilaterally with no reported risk factor for progressive or late-onset sensorineural hearing loss (SNHL).

The plan also included cardiac, ophthalmic, and neurological examinations. The patient was sent for a cardiac examination to exclude an atrial septal defect and pulmonary stenosis. An echocardiogram revealed no cardiac disease or atrial septal defect, or residual pulmonary stenosis. The electrocardiogram showed a normal sinus rhythm. Moreover, there was no sign of pericardial effusion. Her chest examination revealed good bilateral air entry. Her last recorded vitals were a blood pressure of 95/53 mmHg, a pulse of 93 times per minute, an axillary temperature of 36.8 °C (98.2 °F), and a respiration rate of 18 times per minute.

The plan also comprised microarray, whole exome sequencing (WES), and brain studies under magnetic resonance imaging (MRI). MRI findings of the head revealed a smaller size of the head, implying microcephaly, and a thickened calvarium with a hyperplastic marrow signal diffusion restriction, diffuse moderate enhancement, and a FLAIR hyperintense marrow signal, signifying marrow hyperplasia. On MRI, there was diffuse, mild cortical volume loss, mildly dilated ventricles, and no midline shift or mass effect. There was also a diffuse FLAIR signal in the cingulate gyrus, extending superiorly into the precuneus bilaterally. Volume loss and abnormally shaped atrophic gyri were seen in the precuneus, especially along the medial cortex, and were best appreciated in axial T2 sequences. Additionally, there were areas of subcortical and juxtacortical white matter. A FLAIR hyperintense signal abnormality was seen in the frontal lobes bilaterally (axial FLAIR series 400/120). There was a focal blurring of gray-white differentiation in the left medial frontal gyrus. There were normal hippocampal formations and fornices, the basal ganglia, the pituitary gland, the corpus callosum, the brainstem, and the cerebellum. Myelination was complete, and there was no sign of acute infarcts, hemorrhagic lesions, or masses. The post-contrast sequences showed no enhancing lesions. Moreover, there was no significantly increased leptomeningeal or dural enhancement. However, there was a mild mucosal thickening in the paranasal sinuses, with predominantly mucosal thickening seen in the right frontal, sphenoid, and maxillary sinuses and ethmoid air cells, implying right ostiomeatal pattern inflammatory sinusitis. MR spectroscopy revealed no abnormal metabolites in the basal ganglia or the white matter. The area of abnormality in the medial frontal cortices also showed no abnormal metabolites. Tandem mass spectrometry was used for the blood's acylcarnitine profile as part of a basic metabolic workup, and the results were unremarkable. Lactate levels were within the normal range (0.5-2.20 mmol/L). Urine for organic acids, plasma amino acids, and liver function tests was normal. Chromosomal microarray (CMA) testing showed a negative result. The whole exome sequence (WES) revealed a heterozygous VUS in the DHX30 gene. DHX30 (NM_138615.2:c.2387C>T):c.2387C>T p.(Pro796Leu) was classified as a variant of uncertain significance (class 3) according to the recommendations of CENTOGENE lab and ACMG. 

The ACMG classification was made according to the following criteria [6]:

Pathogenic Moderate (PM1): UniProt protein DHX30_HUMAN domain 'Helicase C-terminal' has 10 missense/in-frame variants (five pathogenic variants, five uncertain variants, and no benign), which qualifies as supporting pathogenic, Pathogenic Moderate( PM2): Extremely low frequency in gnomAD population databases, Pathogenic Supporting(PP2): Missense variant in a gene with a low rate of benign missense mutations and for which missense mutation is a common mechanism of a disease and Pathogenic Supporting(PP3): Multiple in silico prediction tools such in BayesDel_addAF = 0.199 is between 0.161 and 0.22 ⇒ supporting pathogenic, align GVGD, a mathematical missense substitution analyzer, predicted C65 class for the variant (DHX30, c.2387C>T p.(Pro796Leu)), implying that the gene mutation is most likely to interfere with function, the Mutation Taster evaluated the mutated gene variant as disease-causing. Moreover, conservation was found to be high.

Then we sent the target gene testing for both asymptomatic parents, which confirmed that mutation is a De novo in the index case but without paternity and maternity confirmed, which will add another Pathogenic Moderate (PM6) to the variant classification. So by having three moderate (PM, PM2 & PM6) and two supporting (PP2&PP3), we can upgrade the classification to likely pathogenic.

## Discussion

RNA helicases play an important role in various aspects of RNA metabolism. The majority of RNA metabolic processes, such as synthesis, nuclear processing and export, translation, RNA storage and decay, and ribonucleoprotein (RNP) assembly, depending on the ATP-dependent unwinding of RNA secondary structures by RNA helicases (RHs) [1]. The gene DHX30 codes for proteins. It is a member of the group of ATP-dependent RNA helicases called DExH-boxes [1]. The DHX30 gene is also found to regulate mitochondrial ribosome assembly and various stages of the RNA life cycle [2,3]. Numerous pathogenic DHX30 variants have been reported. Based on the clinical course, the location and kind of variation are two established clinical subtypes. While mutations in helicase core motifs (HCM) result in a severe phenotype, DHX30 loss-of-function mutations induce a milder phenotype. HCM missense variants that cause a harmful gain-of-function with regard to the formation of stress granule SG in addition to the loss of ATPase and helicase activity [5]. Individuals with HCM heterozygous missense variations exhibit gait problems, significant speech impairment, developmental delay, and intellectual incapacity [5].

The autosomal dominant disorder NEDMIAL, a unique neurodevelopmental disorder with severe motor impairment and nonexistent language, is characterized by profoundly delayed psychomotor development. Patients who are affected display muscle hypotonia, eating problems, an ataxic gait, or a lack of capacity to walk. Their capacity to develop cognitively is severely limited. A significant intellectual handicap is a constant, and speech development is minimal or nonexistent. Additionally, it exhibits atypical behaviors such as autistic traits, a low threshold for frustration, hand flapping, and stereotypes. The presence of sleep difficulties, generalized facial dysmorphism, and joint hypermobility may also be present [4]. Lessel et al.'s groundbreaking research in this area revealed NEDMIAL in 12 people and revealed six unique de novo heterozygous missense variants in the DHX30 gene [1]. The first case of two siblings with the same missense variant of the DHX30 gene who also had adducted thumbs, cryptorchidism, and gonadal mosaicism was reported by Cross et al. [7].

According to a study by Mannucci et al., all 19 of the heterozygous missense variant carriers in the helicase core domain exhibit global developmental delay (GDD), intellectual disability (ID), severe verbal impairment, and aberrant gait patterns. More specifically, all of the people had intellectual disabilities, and just nine (47%) of them had ever learned to walk. Their gaits were all ataxic. Seventy-four percent of the group did not speak at all, four (21%) spoke only one word, and only one (a mosaic for the de novo p.(Ala734Asp) variant) made simple sentences. Additional phenotypic characteristics included muscular hypotonia in 18 (95%) cases, feeding issues in 16 (84%), microcephaly in 13 (81%), joint hypermobility in 14 (74%), structural brain anomalies in 11 (65%), sleep issues in nine, strabismus in eight, autistic features in five, and seizures in four (21%) [5].

Miyaki et al. presented two cases with abnormal variants of DHX30, one (p.Ser737Phe) with impaired RNA helicase activity and both (p.Ser737Phe, p.Arg782Gln) with decreased ATPase activity. Both patients had symptoms that are similar to those of DHX30-related disease, including microcephaly, profound developmental delay, hypotonia, speech impairment, no independent walking, autistic behavior, stereotypical movements, scoliosis, cerebral atrophy, dilated ventricles, and delayed myelination [8]. A Japanese adult (22 years old) with a unique missense variant and two girls with de novo missense mutations in DHX30 were reported by Ueda et al. [4].

A 12-year-old girl presented to our hospital with the typical manifestations of DHX30 mutations. The diagnosis of NEDMIAL is difficult and requires whole exome sequencing (WES) or magnetic resonance imaging (MRI) [1]. Numerous examinations and a basic lab workup were done to exclude other possible causes of the presenting complaints of severe motor developmental delay, intellectual disability, speech impairment, and gait abnormalities. The presentation of the DHX30 mutation resembles that of Rett syndrome and Angelman syndrome [9]. The clinical findings of our case are compared to the clinical findings of cases reported by Lessel et al. [1] and Cross et al. [7] and presented in Table 1.

**Table 1:** A comparison of the clinical findings of our case and the clinical findings of cases reported by Lessel et al. and Cross et al.

Sex Clinical findings	Patient (this case report)	[ 14previously reported cases (Lessel et al. [1], Cross et al. [7]	
				Female	6 Males, 8 Females	
Age at last examination	12 years	3-17 years, 6 months, 15 months	
Intellectual disability	YES	14 YES	
Speech ability	Non-verbal	20 words, 4 words, 11 non-verbal, 1 N/A	
Motor development delay	YES	14/14 YES	
Muscular hypotonia	YES	14/14 YES	
Seizure	YES	3/14 YES	
Feeding difficulties	YES	11/14 YES	
Nystagmus	YES	6/14 YES	
Joint hypermobility	YES	6/14 YES	
Brain MRI anomalies	YES	10/14 YES	
Unilateral cryptorchidism	NO	4/6 YES	
DHX30 alteration	c.2387C>T, p.(Pro796Leu)	c.1478 G>A, p.(Arg493His) (2/14), c.1685A>G, p.(His562Arg) (1/14), c.2093 C>T, p.(Ser698Phe) (2/14) c.2342 G>A, p.(Gly781Asp) (2/14), c.2344 C>T, p.(Arg782Trp) (3/14), c.2353 C>T, p.(Arg785Cys) (3/14), c.2354 G>A, p.(Arg785His) (1/14)	

## Conclusions

To conclude, we reported a case of neurodevelopmental disorder with severe motor impairment and absent language (NEDMIAL) with a De novo novel DHX30 mutation detected by the whole exome sequence. Additionally, we suggested upgrading the variant classification of DHX30:p.Pro796Leu to likely pathogenic, according to the evidence found in our patient. To the best of our knowledge, this is the first reported case of this mutation and disorder in the Middle East.
